# Supplementary material for: Ablation of STAT3 in Purkinje cells reorganizes cerebellar synaptic plasticity in long-term fear memory network
Source: eLife. 2021 Jan 18;10:e63291. doi: 10.7554/eLife.63291 (PMC7813544; doi:10.7554/eLife.63291)
Supplement: Supplementary file 1. [file elife-63291-supp1.docx]

**Supplementary file 1**

**Supplementary Table 1 List of up/down genes from analysis of KEGG pathway**

Kyoto Encyclopedia of Genes and Genomes (KEGG) pathway has been applied to RNA-seq differential expression analyses.

| **log(pval)** | **KEGG pathway, upgenes** |  | **log(pval)** | **KEGG pathway, down genes** |
| --- | --- | --- | --- | --- |
| 22.26281 | Alzheimer's disease |  | 4.017729 | Phototransduction |
| 18.08619 | Parkinson's disease |  | 2.165579 | Neuroactive ligand-receptor interaction |
| 18.03716 | Oxidative phosphorylation |  |  |  |
| 14.66555 | Huntington's disease |  |  |  |
| 10.31785 | Non-alcoholic fatty liver disease (NAFLD) |  |  |  |
| 10.14388 | Retrograde endocannabinoid signaling |  |  |  |
| 8.869666 | Synaptic vesicle cycle |  |  |  |
| 8.580044 | Dopaminergic synapse |  |  |  |
| 8.291579 | Ribosome |  |  |  |
| 7.019088 | Adrenergic signaling in cardiomyocytes |  |  |  |
| 6.962574 | Cardiac muscle contraction |  |  |  |
| 6.419075 | Long-term depression |  |  |  |
| 5.524329 | GABAergic synapse |  |  |  |
| 5.374688 | Long-term potentiation |  |  |  |
| 5.179799 | Glutamatergic synapse |  |  |  |
| 5.175224 | Citrate cycle (TCA cycle) |  |  |  |
| 5.084073 | Endocrine and other factor-regulated calcium reabsorption |  |  |  |
| 4.899629 | Thyroid hormone signaling pathway |  |  |  |
| 4.752027 | cGMP-PKG signaling pathway |  |  |  |
| 4.716699 | Carbon metabolism |  |  |  |
| 4.182435 | Endocytosis |  |  |  |
| 3.742321 | Oxytocin signaling pathway |  |  |  |
| 3.514279 | Sphingolipid signaling pathway |  |  |  |
| 3.293282 | Amphetamine addiction |  |  |  |
| 3.291579 | Ubiquitin mediated proteolysis |  |  |  |
| 3.273273 | cAMP signaling pathway |  |  |  |
| 3.109579 | Autophagy - animal |  |  |  |
| 3.094744 | Phosphatidylinositol signaling system |  |  |  |
| 3.067526 | Biosynthesis of amino acids |  |  |  |
| 3.013676 | Glucagon signaling pathway |  |  |  |
| 2.970616 | Salivary secretion |  |  |  |
| 2.847712 | Oocyte meiosis |  |  |  |
| 2.707744 | Insulin secretion |  |  |  |
| 2.694649 | Gastric acid secretion |  |  |  |
| 2.517126 | Choline metabolism in cancer |  |  |  |
| 2.333482 | Circadian entrainment |  |  |  |
| 2.117475 | Fc gamma R-mediated phagocytosis |  |  |  |
| 2.091515 | Metabolic pathways |  |  |  |
| 2.06956 | Ferroptosis |  |  |  |
| 1.931814 | Estrogen signaling pathway |  |  |  |
| 1.876148 | Collecting duct acid secretion |  |  |  |
| 1.707744 | Gap junction |  |  |  |
| 1.642065 | 2-Oxocarboxylic acid metabolism |  |  |  |
| 1.595166 | Mitophagy - animal |  |  |  |
| 1.531653 | Pancreatic secretion |  |  |  |
| 1.496209 | Calcium signaling pathway |  |  |  |
| 1.413413 | Salmonella infection |  |  |  |
| 1.376751 | Inositol phosphate metabolism |  |  |  |
| 1.374688 | Focal adhesion |  |  |  |
